# Engaging social activities prevent stroke and myocardial infraction by raising awareness of warning symptoms: A cross-sectional survey study

**DOI:** 10.3389/fpubh.2022.1043875

**Published:** 2023-01-16

**Authors:** Gahyeon Kim, Hyeokjoo Jang, Sebin Kwon, Bumyeol Lee, Suk-Yong Jang, Wonjeong Chae, Sung-In Jang

**Affiliations:** ^1^Medical School, Yonsei University College of Medicine, Seoul, Republic of Korea; ^2^Department of Healthcare Management, Graduate School of Public Health, Yonsei University, Seoul, Republic of Korea; ^3^Institute of Health Services Research, Yonsei University, Seoul, Republic of Korea; ^4^Department of Health Policy and Management, Graduate School of Public Health, Yonsei University, Seoul, Republic of Korea; ^5^Department of Preventive Medicine, Yonsei University, Seoul, Republic of Korea

**Keywords:** social activity, cardiovascular disease, stroke, myocardial infarction, self-awareness, prevention

## Abstract

**Background:**

Stroke and myocardial infarction (MI) are medical emergencies, and early treatment within the golden hour is crucial for good prognosis. Adequate knowledge about the warning symptoms can shorten the onset-to-door time. Various factors affect the level of awareness, including social activity. This study aimed to determine if engaging in social activity is associated with the awareness of the warning symptoms of stroke and MI.

**Methods:**

This cross-sectional study analyzed 451,793 participants from the 2017 and 2019 Korea Community Health Survey. Based on five questions for each of stroke and MI symptoms, participants were divided into an awareness group (replied “Yes” to all five questions) and unawareness group. Engagement in social activities (i.e., religious, friendship, leisure, and volunteer activity) was evaluated through a questionnaire. Multiple logistic regression analysis was performed to evaluate the relation between social activity and awareness of warning symptoms.

**Results:**

Overall, 52.6% participants were aware of the warning symptoms of stroke, and 45.8% of MI. Regular engagement in at least one social activity, particularly friendship or volunteer activity, was associated with better awareness of the warning symptoms, both stroke (OR: 1.21, 95% CI: 1.20–1.23) and MI (OR: 1.22, 95% CI: 1.20–1.24). Additionally, more diverse types of social activities were associated with higher levels of awareness. Relationship between social activity and awareness showed positive association with participants older than 60 years, rural residents, or with low socioeconomic status.

**Conclusion:**

Engagement in social activity was significantly associated with better knowledge about the warning symptoms of stroke and MI. For early hospital treatment after symptom onset, participation in social activities could be beneficial.

## Highlights

- The golden hour care for stroke and myocardial infarction is essential that can impact its prognosis.
- Regular engagement in at least one social activity was associated with better awareness of the warning symptoms of stroke and MI.
- Engagement in social activity was significantly associated with better knowledge about stroke and MI warning symptoms that can lead to early detection and prevention.

## Introduction

Cardiovascular diseases (CVDs) are a group of disorders of the heart and blood vessels, such as stroke and myocardial infarction (MI). According to the World Health Organization (WHO), an estimated 17.9 million people died from CVDs in 2019 worldwide, accounting for 32% of the total global deaths. In particular, 85% of these deaths were due to stroke and MI, which are therefore considered major public health concerns worldwide (1). In South Korea, CVD is the second leading cause of death after cancer. In 2020, there were about 27.4 and 42.6 deaths per 100,000 people from cerebrovascular disease (i.e., stroke) and ischemic heart disease (i.e., MI), respectively (2). Given that the population is rapidly aging and the risk factors for CVDs are increasing, the socioeconomic burden of these diseases continues to remain high (3).

Ischemic stroke and MI occur when the blood supply to the brain or heart muscle is obstructed. It can rapidly cause irreversible tissue damage, which can lead to serious complications and even death (4). Therefore, stroke and MI are medical emergencies, and urgent interventions such as thrombolysis or endovascular procedure are required. In fact, it has been shown that early treatment after the onset of symptoms is crucial for a better prognosis (5–7). However, according to Korean national statistics in 2020, only 41.4% of stroke patients and 44.0% of MI patients arrived at the hospital within 3 h after the onset of symptoms. Moreover, only 18.5 and 18.6% of stroke and MI patients, respectively, arrived within 1 h, the so-called golden hour (8). This is partially due to a lack of knowledge about the warning symptoms of stroke and MI, not only among the patients themselves but also among the surrounding people. Previous studies have shown that the better a person recognizes symptoms, the faster he or she presents for emergency treatment (9–11). Accordingly, the Korean government has made efforts through health projects to raise public awareness regarding the warning symptoms of CVDs since 2018 (12).

Previous studies have reported various factors that are associated with the level of knowledge about the warning symptoms of CVDs, including sex, age, race, region, marital status, education level, income, occupation, smoking, drinking, physical activity, and comorbidities (13–21). In fact, there exist several routes for people to learn about the warning symptoms of CVDs, for example through family members, mass media, school class, workplace, and hospital visit. Considering that the population is aging and one-person households are becoming more common these days, social activities such as religious activities or volunteer work can also be useful channels to obtain such knowledge (22).

Although there have been studies on the awareness of warning symptoms of CVDs among Koreans, no study has evaluated its relationship to social activities. Public health projects promoting social activities may be effective in reducing serious complications following stroke and MI if they can help in increasing awareness of the warning symptoms of CVDs. Thus, we aimed to determine if engagement in social activities is associated with increased awareness of the warning symptoms of stroke and MI. Furthermore, we also assessed if the awareness differs by the type and number of social activities, using a representative nationwide health survey.

## Methods

### Study population and data

The study data were obtained from the Korea Community Health survey (KCHS) conducted in 2017 and 2019. This nationwide survey has been conducted annually by the Korea Disease Control and Prevention Agency (KCDC) since 2008. The dataset is publicly available on the KCHS's official website (https://chs.kdca.go.kr/chs/). Multistage, stratified sampling methods were used to select representative households, and trained surveyors carried out personal interviews with adults aged 19 years or older living in these households. The KCHS questionnaire consists of ~200 items in 20 fields including demographics, health-related behaviors, morbidities, and social environments.

A total of 228,381 people participated in the 2017 KCHS and 229,099 people in 2019. Among them, we excluded individuals who refused to respond to the questions on the warning symptoms of stroke and MI or to the questions on social activities. Participants with missing data on any variables used in this study were also excluded. Finally, 451,793 participants were included in the analysis.

### Variables

The dependent variable was defined as the level of awareness of the warning symptoms of stroke and MI. To evaluate awareness of the warning symptoms of stroke, participants were asked the following questions: “If you think that the following are the symptoms of stroke, answer ‘Yes,' or if you do not think so, answer ‘No.' Please answer ‘I do not know' if you are unsure.” Warning symptoms included the following: (1) sudden numbness or weakness of the face, arm, or leg on one side of the body; (2) sudden difficulty in speaking or understanding speech; (3) sudden visual impairment of one or both eyes, or double vision; (4) sudden dizziness or loss of balance; (5) sudden severe headache never experienced before. These are the five cardinal symptoms of stroke, which are well-established for assessing stroke awareness (9). Only participants who replied “Yes” to all five questions were considered to have awareness of the warning symptoms of stroke, based on the definition by the Korean Disease Control and Prevention Agency (KDCA) (23). Participants who replied “No” or “I do not know” to at least one of the five questions were categorized as the unaware group. The questions for evaluating awareness of the warning symptoms of MI were the same as those for stroke awareness. According to the United States Centers for Disease Control and Prevention (CDC), the five symptoms included the following: (a) sudden pain or discomfort in the jaw, neck, or back; (b) sudden weakness, dizziness, nausea, or cold sweating; (c) sudden pain, pressure, or discomfort in the chest; (d) sudden pain or discomfort in the arm or shoulder (e) sudden shortness of breath (24). Participants who answered “Yes” to all five questions were assigned to the awareness group based on the KDCA definition and results of previous studies (15, 21, 23).

The main variable of interest was engagement in social activity, which was defined on the basis of the following four questions. Participants were asked “Do you regularly engage in religious activities at least once a month?” and the same question was asked also on friendship, leisure, and volunteer activities. Individuals who replied “Yes” to at least one of the four questions were assigned to the “social activity group,” while those who replied “No” to all four were assigned to the “no social activity group.”

This study included the potential confounding variables as covariates: sex, age, region, marital status, household type, monthly household income, level of education, occupation, smoking, drinking, and comorbidities. The year of examination was also adjusted. Household types were divided based on the number of cohabiting generations, in which individuals living alone were classified into one generation group. Monthly household income was categorized into following three groups: low (<200 × 10^4^ KRW), middle (200–400 × 10^4^ KRW), and high (≥400 × 10^4^ KRW). We classified the participants' occupations into white-collar worker (managers, professionals, clerks, and service and sale workers), blue-collar worker (agricultural, forestry and fishery workers, mechanical and manual laborers, and armed forces), and unemployed (housewives, students, and unemployed). Smoking status was categorized into never, current and ex-smoker. Those who smoked <100 cigarettes in their lifetime were classified as non-smokers. Alcohol consumption was divided based on the frequency of drinking over the past year. Comorbidities included hypertension and diabetes.

### Statistical analysis

All analyses were done separately for stroke and MI. The frequencies and proportions of independent variables were compared using the chi-square test to confirm the differences between the individuals who were aware of the warning symptoms and those who are not. We performed multivariate logistic regression analysis after adjusting for demographics, socioeconomic status, comorbidities, and the examination year to identify the factors that are associated with an awareness of the warning symptoms. The results are shown as odds ratios (ORs) and 95% confidential intervals (95% CIs). In addition, each factor (i.e., type and number) of social activity was also analyzed through multiple logistic regression analysis to determine which factor was the most relevant to the awareness of the warning symptoms. Finally, we conducted subgroup analyses stratified by other covariates. All statistical analyses were conducted using SAS software (version 9.4, SAS Institute, Cary, NC, USA), and *p*-value < 0.05 was considered statistically significant.

## Results

General characteristics of the study population are described in Table 1. Of the total 451,793 participants, 237715 (52.6%) were aware of all five warning symptoms of stroke and 206,743 (45.8%) of those of MI. A total of 319,796 (70.8%) participants regularly engaged in social activities at least once a month. Among them, 177,374 (55.5%) recognized stroke symptoms and 154,461 (48.3%) recognized MI symptoms. In the group not engaging in social activity, the number of participants who recognized stroke and MI symptoms were 60,341 (45.7%) and 52,282 (39.6%), respectively.

**Table 1:** General characteristics of study population.

	**Total**		**Aware of warning symptoms of stroke**			**Aware of warning symptoms of MI**		
	***N***	**%**	***n***	**%**	***p*-value**	***n***	**%**	***p*-value**
**Total**	451,793	(100.0)	237,715	(52.6)		206,743	(45.8)	
**Social activity**					<0.0001			<0.0001
Yes	319,796	(70.8)	177,374	(55.5)		154,461	(48.3)	
No	131,997	(29.2)	60,341	(45.7)		52,282	(39.6)	
**Sex**					<0.0001			<0.0001
Male	202,519	(44.8)	102,903	(50.8)		90,727	(44.8)	
Female	249,274	(55.2)	134,812	(54.1)		116,016	(46.5)	
**Age**					<0.0001			<0.0001
19–29	47,097	(10.4)	19,972	(42.4)		19,370	(41.1)	
30–39	56,007	(12.4)	29,434	(52.6)		26,879	(48.0)	
40–49	74,470	(16.5)	42,813	(57.5)		37,181	(49.9)	
50–59	88,030	(19.5)	52,661	(59.8)		44,899	(51.0)	
60–69	84,655	(18.7)	48,476	(57.3)		41,087	(48.5)	
≥70	101,534	(22.5)	44,359	(43.7)		37,327	(36.8)	
**Region**					<0.0001			<0.0001
Capital area	144,209	(31.9)	74,354	(51.6)		64,865	(45.0)	
Urban	70,506	(15.6)	37,518	(53.2)		33,285	(47.2)	
Rural	237,078	(52.5)	125,843	(53.1)		108,593	(45.8)	
**Marital status**					<0.0001			<0.0001
Married	302,800	(67.0)	170,735	(56.4)		146,991	(48.5)	
Divorced, widowed, or separated	79,446	(17.6)	36,054	(45.4)		30,614	(38.5)	
Never married	69,547	(15.4)	30,926	(44.5)		29,138	(41.9)	
**Household type**					<0.0001			<0.0001
1 generation	213,039	(47.2)	110,220	(51.7)		94,652	(44.4)	
2 generations	206,473	(45.7)	110,770	(53.7)		97,359	(47.2)	
3 or more generations	32,281	(7.2)	16,725	(51.8)		14,732	(45.6)	
**Monthly household income**					<0.0001			<0.0001
Low	151,814	(33.6)	71,303	(47.0)		60,930	(40.1)	
Middle	140,412	(31.1)	75,462	(53.7)		66,152	(47.1)	
High	159,567	(35.3)	90,950	(57.0)		79,661	(49.9)	
**Highest level of education**					<0.0001			<0.0001
Elementary school and below	107,963	(23.9)	46,668	(43.2)		39,410	(36.5)	
Middle school	51,835	(11.5)	27,717	(53.5)		23,496	(45.3)	
High school	128,515	(28.5)	71,695	(55.8)		61,581	(47.9)	
College and above	163,480	(36.2)	91,635	(56.1)		82,256	(50.3)	
**Occupation**					<0.0001			<0.0001
White-collar worker	145,556	(32.2)	84,167	(57.8)		74,763	(51.4)	
Blue-collar worker	137,857	(30.5)	70,141	(50.9)		60,200	(43.7)	
Unemployed	168,380	(37.3)	83,407	(49.5)		71,780	(42.6)	
**Smoking**					<0.0001			<0.0001
Never smoker	291,135	(64.4)	156,933	(53.9)		136,007	(46.7)	
Ex-smoker	84,468	(18.7)	44,478	(52.7)		38,160	(45.2)	
Current smoker	76,190	(16.9)	36,304	(47.7)		32,576	(42.8)	
**Drinking**					<0.0001			<0.0001
None	155,551	(34.4)	80,291	(51.6)		69,015	(44.4)	
≤ 1 time per month	110,123	(24.4)	59,707	(54.2)		51,694	(46.9)	
2–4 times per month	89,799	(19.9)	48,128	(53.6)		42,417	(47.2)	
2–3 times per week	62,936	(13.9)	33,090	(52.6)		29,320	(46.6)	
≥4 times per week	33,384	(7.4)	16,499	(49.4)		14,297	(42.8)	
**Diagnosed with hypertension**					<0.0001			<0.0001
Yes	126,624	(28.0)	65,514	(51.7)		55,259	(43.6)	
No	325,169	(72.0)	172,201	(53.0)		151,484	(46.6)	
**Diagnosed with diabetes**					<0.0001			<0.0001
Yes	51,071	(11.3)	25,748	(50.4)		22,133	(43.3)	
No	400,722	(88.7)	211,967	(52.9)		184,610	(46.1)	
**Examination year**					<0.0001			<0.0001
2017	225,383	(49.9)	110,437	(49.0)		95,651	(42.4)	
2019	226,410	(50.1)	127,278	(56.2)		111,092	(49.1)	

Table 2 shows the factors associated with the awareness of warning symptoms of stroke and MI. After adjusting for covariates, people who participated in social activities were more likely to have awareness of the warning signs than people who did not. These results were significant for both stroke (OR: 1.21, 95% CI: 1.20–1.23) and MI (OR: 1.22, 95% CI: 1.20–1.24). Moreover, women (stroke, OR: 1.19, 95% CI: 1.16–1.21; MI, OR: 1.11, 95% CI: 1.09–1.13), married individuals (stroke, OR: 1.27, 95% CI: 1.24–1.30; MI, OR: 1.22, 95% CI: 1.19–1.25), and highly educated individuals with college or above educational level (stroke, OR: 2.06, 95% CI: 2.01–2.11; MI, OR: 1.84, 95% CI: 1.80–1.89) showed a significantly positive level of awareness. On the contrary, current smoking (stroke, OR: 0.82, 95% CI: 0.81–0.84; MI, OR: 0.87, 95% CI: 0.85–0.88) and drinking more than 4 times per week (stroke, OR: 0.91, 95% CI: 0.88–0.93; MI, OR: 0.90, 95% CI: 0.88–0.93) was associated with low knowledge of stroke and MI symptoms.

**Table 2:** Factors associated with awareness of warning symptoms of stroke and MI.

	**Aware of warning symptoms of stroke**			**Aware of warning symptoms of MI**		
	**OR^a^**	**95% CI**	***p*-value**	**OR^a^**	**95% CI**	***p*-value**
**Social activity**						
Yes	1.21	(1.20–1.23)	<0.0001	1.22	(1.20–1.24)	<0.0001
No	1.00	-		1.00	-	
**Sex**						
Male	1.00	-		1.00	-	
Female	1.19	(1.16–1.21)	<0.0001	1.11	(1.09–1.13)	<0.0001
**Age**						
19–29	1.00	-		1.00	-	
30–39	1.29	(1.25–1.33)	<0.0001	1.15	(1.12–1.19)	<0.0001
40–49	1.59	(1.54–1.64)	<0.0001	1.25	(1.22–1.29)	0.0157
50–59	1.92	(1.86–1.99)	<0.0001	1.42	(1.38–1.47)	<0.0001
60–69	2.10	(2.02–2.17)	<0.0001	1.51	(1.45–1.56)	<0.0001
≥70	1.50	(1.45–1.56)	0.2442	1.12	(1.08–1.17)	<0.0001
**Region**						
Capital area	1.00	-		1.00	-	
Urban	1.09	(1.07–1.11)	0.1886	1.11	(1.09–1.13)	0.0038
Rural	1.21	(1.19–1.23)	<0.0001	1.18	(1.17–1.20)	<0.0001
**Marital status**						
Married	1.27	(1.24–1.30)	<0.0001	1.22	(1.19–1.25)	<0.0001
Divorced, widowed, or separated	1.00	(0.97–1.03)	<0.0001	1.00	(0.97–1.03)	<0.0001
Never married	1.00	-		1.00	-	
**Household type**						
1 generation	1.02	(1.00–1.05)	0.0082	1.00	(0.97–1.02)	0.1041
2 generations	1.00	(0.97–1.02)	0.0866	0.97	(0.95–0.99)	0.0001
3 or more generations	1.00	-		1.00	-	
**Monthly household income**						
Low	1.00	-		1.00	-	
Middle	1.06	(1.04–1.07)	0.3704	1.04	(1.03–1.06)	0.0076
High	1.10	(1.08–1.12)	<0.0001	1.05	(1.03–1.07)	0.0002
**Highest level of education**						
Elementary school and below	1.00	-		1.00	-	
Middle school	1.38	(1.35–1.41)	<0.0001	1.30	(1.27–1.33)	<0.0001
High school	1.70	(1.66–1.73)	<0.0001	1.52	(1.49–1.56)	<0.0001
College and above	2.06	(2.01–2.11)	<0.0001	1.84	(1.80–1.89)	<0.0001
**Occupation**						
White-collar worker	1.14	(1.13–1.16)	<0.0001	1.16	(1.14–1.18)	<0.0001
Blue-collar worker	1.00	(0.98–1.02)	<0.0001	1.00	(0.98–1.01)	<0.0001
**Smoking**						
Never smoker	1.00	-			-	
Ex-smoker	0.95	(0.93–0.97)	<0.0001	0.94	(0.92–0.96)	0.2301
Current smoker	0.82	(0.81–0.84)	<0.0001	0.87	(0.85–0.88)	<0.0001
**Drinking**						
None	1.00	-			-	
≤ 1 time per month	0.98	(0.97–1.00)	<0.0001	0.96	(0.94–0.98)	0.2159
2–4 times per month	0.97	(0.95–0.99)	0.0866	0.95	(0.94–0.97)	0.7106
2–3 times per week	0.94	(0.92–0.96)	0.0058	0.95	(0.93–0.97)	0.3064
≥4 times per week	0.91	(0.88–0.93)	<0.0001	0.90	(0.88–0.93)	<0.0001
**Diagnosed with hypertension**						
Yes	1.09	(1.08–1.11)	<0.0001	1.05	(1.04–1.07)	<0.0001
No	1.00	-		1.00	-	
**Diagnosed with diabetes**						
Yes	0.96	(0.94–0.98)	<0.0001	1.00	(0.98–1.02)	0.7631
No	1.00	-		1.00	-	
**Examination year**						
2017	1.00	-		1.00	-	
2019	1.33	(1.32–1.35)	<0.0001	1.31	(1.29–1.32)	<0.0001

^a^Adjusted for social activity, sex, age, region, marital status, household type, monthly household income, highest level of education, occupation, smoking, drinking, hypertension, diabetes, and examination year.

**Figure 1:**
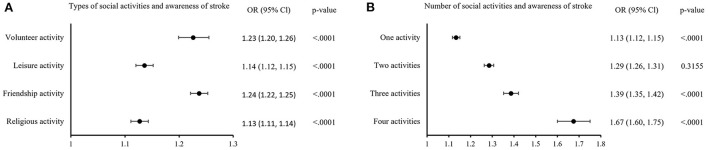
Subgroup analysis of social activities and awareness of warning symptoms of stroke. **(A)** Shows the odds of having awareness in individuals who participate in each types of social activities compared to who do not. **(B)** Shows the odds of having awareness depending on the number of social activities. Odds ratio were adjusted for sex, age, region, marital status, household type, monthly household income, highest level of education, occupation, smoking, drinking, hypertension, diabetes, and examination year.

**Figure 2:**
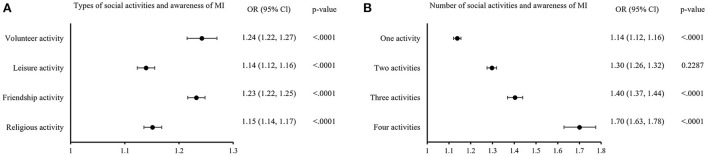
Subgroup analysis of social activities and awareness of warning symptoms of MI. **(A)** Shows the odds of having awareness in individuals who participate in each types of social activities compared to who do not. **(B)** Shows the odds of having awareness depending on the number of social activities. Odds ratio were adjusted for sex, age, region, marital status, household type, monthly household income, highest level of education, occupation, smoking, drinking, hypertension, diabetes, and examination year.

The associations between social activities and awareness of warning symptoms of stroke and MI are shown in Figures 1, 2. Regarding the types of social activities, people who were regularly involved in friendship activity (stroke, OR: 1.24, 95% CI: 1.22–1.25; MI, OR: 1.23, 95% CI: 1.22–1.25) or volunteer activity (stroke, OR: 1.23, 95% CI: 1.20–1.26; MI, OR: 1.24, 95% CI: 1.22–1.27) were the most likely to be aware of stroke and MI symptoms. In addition, the more diverse the types of social activities, the higher the odds of having awareness of stroke and MI symptoms. Especially, people who participated in all four types of social activities had a significantly better level of awareness (stroke, OR: 1.67, 95% CI: 1.60–1.75; MI, OR: 1.70, 95% CI: 1.63–1.78) compared to people who participated in none of them.

Table 3 represents the subgroup analysis stratified by other independent variables. In the oldest age group of 70 years or older, individuals who were involved in social activities were significantly more likely to be aware of the warning symptoms of stroke (OR: 1.33, 95% CI: 1.29–1.36) and MI (OR: 1.34, 95% CI: 1.30–1.38). Among participants who were divorced, widowed, or separated, engagement in social activities was significantly associated with the awareness of warning symptoms (stroke, OR: 1.28, 95% CI: 1.24–1.32; MI, OR: 1.28, 95% CI: 1.24–1.32). Furthermore, the association between social activities and awareness of warning symptoms was especially significant in the group with low household income, poor education, or unemployed status.

**Table 3:** Subgroup analysis of the association between social activities and awareness of warning symptoms of stroke and MI stratified by covariates.

	**Aware of warning symptoms of stroke**			**Aware of warning symptoms of MI**		
	**Social activity**			**Social activity**		
	**No**	**Yes**		**No**	**Yes**	
	**OR**	**OR^a^**	**CI**	**OR**	**OR^a^**	**CI**
**Sex**						
Male	1.00	1.21	(1.18–1.23)	1.00	1.24	(1.22–1.27)
Female	1.00	1.22	(1.20–1.24)	1.00	1.21	(1.19–1.23)
**Age**						
19–29	1.00	1.15	(1.11–1.19)	1.00	1.17	(1.12–1.21)
30–39	1.00	1.11	(1.07–1.15)	1.00	1.13	(1.09–1.17)
40–49	1.00	1.17	(1.13–1.21)	1.00	1.19	(1.15–1.23)
50–59	1.00	1.19	(1.15–1.23)	1.00	1.20	(1.16–1.24)
60–69	1.00	1.21	(1.17–1.25)	1.00	1.21	(1.17–1.25)
≥70	1.00	1.33	(1.29–1.36)	1.00	1.34	(1.30–1.38)
**Region**						
Capital area	1.00	1.11	(1.08–1.13)	1.00	1.12	(1.10–1.15)
Urban	1.00	1.21	(1.17–1.25)	1.00	1.21	(1.17–1.26)
Rural	1.00	1.27	(1.25–1.30)	1.00	1.28	(1.25–1.30)
**Marital status**						
Married	1.00	1.21	(1.18–1.23)	1.00	1.22	(1.20–1.24)
Divorced, widowed, or separated	1.00	1.28	(1.24–1.32)	1.00	1.28	(1.24–1.32)
Never married	1.00	1.16	(1.12–1.20)	1.00	1.16	(1.13–1.20)
**Household type**						
1 generation	1.00	1.23	(1.20–1.25)	1.00	1.24	(1.22–1.27)
2 generations	1.00	1.19	(1.16–1.21)	1.00	1.20	(1.17–1.22)
3 or more generations	1.00	1.26	(1.20–1.32)	1.00	1.27	(1.21–1.34)
**Monthly household income**						
Low	1.00	1.26	(1.23–1.29)	1.00	1.28	(1.25–1.31)
Middle	1.00	1.21	(1.18–1.24)	1.00	1.21	(1.18–1.24)
High	1.00	1.16	(1.13–1.19)	1.00	1.16	(1.13–1.19)
**Highest level of education**						
Elementary school and below	1.00	1.31	(1.27–1.34)	1.00	1.33	(1.30–1.37)
Middle school	1.00	1.24	(1.19–1.29)	1.00	1.23	(1.18–1.29)
High school	1.00	1.18	(1.15–1.21)	1.00	1.19	(1.16–1.22)
College and above	1.00	1.14	(1.11–1.17)	1.00	1.15	(1.12–1.18)
**Occupation**						
White-collar worker	1.00	1.17	(1.14–1.20)	1.00	1.16	(1.13–1.19)
Blue-collar worker	1.00	1.20	(1.18–1.23)	1.00	1.23	(1.20–1.26)
Unemployed	1.00	1.24	(1.21–1.26)	1.00	1.25	(1.22–1.28)
**Smoking**						
Never smoker	1.00	1.22	(1.20–1.24)	1.00	1.22	(1.20–1.24)
Ex-smoker	1.00	1.21	(1.17–1.25)	1.00	1.27	(1.23–1.31)
Current smoker	1.00	1.17	(1.14–1.21)	1.00	1.20	(1.16–1.24)
**Drinking**						
None	1.00	1.27	(1.24–1.30)	1.00	1.28	(1.25–1.31)
≤ 1 time per month	1.00	1.20	(1.17–1.23)	1.00	1.21	(1.18–1.25)
2–4 times per month	1.00	1.15	(1.11–1.18)	1.00	1.15	(1.12–1.19)
2–3 times per week	1.00	1.16	(1.12–1.20)	1.00	1.17	(1.12–1.21)
≥4 times per week	1.00	1.19	(1.14–1.26)	1.00	1.23	(1.17–1.30)
**Diagnosed with hypertension**						
Yes	1.00	1.24	(1.21–1.27)	1.00	1.27	(1.24–1.31)
No	1.00	1.20	(1.18–1.22)	1.00	1.20	(1.18–1.22)
**Diagnosed with diabetes**						
Yes	1.00	1.21	(1.16–1.26)	1.00	1.26	(1.21–1.31)
No	1.00	1.21	(1.19–1.23)	1.00	1.22	(1.20–1.24)
**Examination year**						
2017	1.00	1.22	(1.19–1.24)	1.00	1.24	(1.21–1.26)
2019	1.00	1.21	(1.19–1.23)	1.00	1.21	(1.18–1.23)

^a^Adjusted for sex, age, region, marital status, household type, monthly household income, highest level of education, occupation, smoking, drinking, hypertension, diabetes, and examination year.

## Discussion

Recognizing the early warning symptoms of CVD can help the patients to obtain emergency treatment within the critical period, which can significantly improve the prognosis and survival (5–7, 9–11). Therefore, raising public awareness of CVDs is an important national issue to reduce the socioeconomic burden of CVDs in the upcoming aging society of Korea. This study was designed to examine if engagement in social activities was related to the awareness of warning symptoms of stroke and MI. Our large population-based analysis revealed that the people who participated in social activities were significantly more likely to have an exact knowledge of the warning symptoms of CVDs compared to people who did not. The odds of association between them were not much different for stroke and MI.

We examined demographic, socioeconomic, and health-related factors to identify other meaningful factors that are related to the awareness of CVD warning symptoms. First, sex was found to be an influential predictor of symptom awareness. Women exhibited higher knowledge than men both for stroke and MI, which is consistent with the results of previous studies (13, 17, 18, 21, 25). Given that the incidence of stroke and MI is higher in men than in women (8), further intervention to raise awareness, especially among men is needed. Regarding the association between age and awareness of CVD symptoms, the results of previous studies varied. Some papers have reported higher awareness in old age (16, 20), while others have revealed the opposite (14, 15, 25). However, most of the studies reported that middle-aged people showed the highest level of awareness. Additionally, in the present study, participants aged 50–69 years had a better knowledge of the warning symptoms compared to the younger and older age groups. This was true for both stroke and MI, but the ORs were higher for stroke compared to MI. This can be explained by the fact that since people over the age of 50 years are at high risk of CVDs (4, 8), they may hear of such experiences from the people around them, and thus tend to be exposed more often to the relevant information. In contrast, young adults lack chances to encounter such situations, and even so, they may not be much interested in health issues. The elderly aged 70 years and older seem to have difficulty in recognizing the warning symptoms due to under education or cognitive impairment (18, 21).

The region of residence was also associated with symptom awareness of stroke and MI, with individuals living in rural areas having higher awareness levels than those living in capital or urban areas. Preceding studies have explained that national projects to spread the knowledge about CVDs may have been more actively promoted in rural areas where emergency treatment is not easily accessible (16, 18). In addition, rural dwellers tend to establish closer connections with their neighbors than city dwellers, which can lead to a higher chance of sharing health knowledge (26). However, further investigations are needed since some studies have also demonstrated contradictory results in that the urban residents knew more about the CVD symptoms (13, 15, 25). Socioeconomic factors such as high household income, high level of education, and white-collar occupations are known predictors of high knowledge of warning symptoms (13–17, 19, 20, 25). This was also seen in our study, both for stroke and MI. People with low socioeconomic status are in a relatively unfavorable condition to acquire health-related information (16), which may result in poor knowledge regarding CVDs.

Smoking and heavy drinking are well-known modifiable risk factors of CVDs (1, 4). In this study, individuals who smoke or drink showed lower awareness of stroke and MI symptoms, which was consistent with previously reported results (13, 16, 20, 21). Hypertension, diabetes, and dyslipidemia also increase the risk of stroke and MI (1, 4), and past studies have revealed that patients with the above underlying diseases had stronger awareness of CVD symptoms (13, 21, 27). This may be because these patients regularly visited doctors and learned about the complications, including stroke and MI (16). In our study, the same results were obtained for hypertension, but not for diabetes.

We further analyzed the association between social activity and awareness about CVD symptoms in terms of the type and number of social activities. Among four types of social activities, friendship and volunteer activities were significantly associated with both stroke and MI awareness. Activities related to friendship and volunteer work tend to allow much time for the participants to engage in conversations with each other (28, 29). During these activities, people build a strong bond, allowing them to share personal matters. This exercise can lead to a higher chance of gaining health-related information and self-awareness on health than people who do not participate in those activities (29–31). Additionally, analysis of the number and types of social activities showed that the more the variety of activities one takes part in, the higher the level of awareness about CVD warning symptoms. In particular, individuals who engaged in all four types of social activities had significantly better knowledge than those who engaged in none of them.

Subgroup analysis revealed that the relationship between social activity and awareness of stroke and MI symptoms differed by demographic and socioeconomic factors. The association was significant in subgroups of individuals aged over 60 years, those living in rural areas, divorced, widowed, separated, or with low socioeconomic status (low household income, low level of education, unemployed). People with these characteristics seem to lack access to other extensive routes to learn about CVD symptoms, such as the internet, family members, school, workplace, or hospital visit (16, 32). This could have resulted in the relatively high emphasis on the role of social activities.

In our study, 52.6 and 45.8% of the overall Korean population had knowledge about the warning symptoms of stroke and MI, respectively. The results show that the awareness of stroke and MI had increased in 2019 from 2017. There was a higher number of individuals who were aware of warning symptoms, and results of logistic analysis exhibited that awareness has increased significantly. From this study, we were not able to seek a specific cause yet; this could be the positive outcome of a continuous national-level health promotion campaign by the government. Since 2013, the Korean government has started health projects to raise awareness of warning symptoms and early management of CVDs. Moreover, comprehensive CVD management plans, regional cardio-cerebrovascular centers, and educational programs were established (12). However, despite such remarkable progress, perception rates are still relatively low compared to 53.0% in the United States (17) and 57.8% in Singapore (33). Together with the rapidly aging population, elderly individuals living alone are emerging as one of the priority groups in Korea (34). These people cannot easily get help from others when a stroke or MI occurs; therefore, being aware of the early warning symptoms is crucial for ensuring proper emergency treatment. Our study results suggest social activity as one of the solutions to reduce the social burden of CVDs in the upcoming aging society. According to our results, social activities are expected to play a significant role, especially among individuals with a low socioeconomic status, dwelling in rural areas, or no longer living with a spouse.

The present study has several strengths. First, this study was based on KCHS, the nationwide representative data of the Korean general population. These data are reliable in that the survey was performed by a national institution. In addition, the large sample size of over total 450,000 participants enabled more precise statistical adjustments. Third, to our knowledge, this is the first study to identify an association between social activities and awareness of warning symptoms of CVDs in Korea. Although previous studies have evaluated some factors related to the awareness of CVD warning symptoms, our research mainly focused on social activities, specifically the type and number of social activities people regularly engage in. Our findings indicate that promoting social activities could also play significant role in shortening the door-to-treatment time in patients with stroke and MI.

However, this study also has some limitations. First, since this was a cross-sectional study, we could not confirm a causal relationship between participation in social activity and awareness of CVD warning symptoms. The possibility of reverse causality cannot be excluded. For example, more health-conscious and, therefore, healthier individuals might tend to participate in more social activities. Further longitudinal studies are needed to clarify this point. Second, variables regarding social activities were self-reported; therefore, recall bias may have been present. Third, we could not evaluate the detailed frequency of social activities because the questionnaire only asked “Do you regularly engage in the activity at least once a month?” Fourth, the knowledge of warning symptoms of stroke and MI was evaluated using closed-ended questions, which are likely to elicit more positive response than open-ended questions. Some studies have proven that closed-ended questions can provide respondents with some indication of what the correct answer could be (35). The questionnaire also did not include any negative or trap questions. This may have resulted in an overestimation of the participants' awareness of the warning symptoms of CVD. Lastly, there may be other behavioral or health-related factors contributing to the level of awareness. For example, the level of exposure to mass media or medical history of CVD may have influenced the knowledge of the warning symptoms of CVD, but these data were unavailable in KCHS. Nonetheless, we included any obtainable factors as covariates, such as a history of hypertension and diabetes which are the main risk factors of CVD (1).

## Conclusion

This study found that engaging in social activity was related to a higher awareness of the Korean population's warning symptoms of stroke and MI. In particular, the association was prominent in people who participated in friendship or volunteer activities and those who were older, lived-in rural areas, or had low socioeconomic status. Stroke and MI are both major health concerns worldwide, and public awareness of the warning symptoms of these diseases is crucial for ensuring early treatment within the golden hour. Therefore, national campaigns and policies for raising awareness of stroke and MI should encourage social activities as the study discovered its association. However, further longitudinal studies are needed to clarify causality.

## Data availability statement

Publicly available datasets were analyzed in this study. This data can be found at: The Korea National Health and Nutrition Examination Survey.

## Ethics statement

Ethical approval was not provided for this study on human participants because, data used for the study is open source data provided by the Government agency for research purpose. Written informed consent for participation was not required for this study in accordance with the national legislation and the institutional requirements.

## Author contributions

GK, WC, and S-IJ designed the study. GK performed the statistical analysis and wrote the original manuscript. WC participated in the analysis. SK, BL, HJ, and S-YJ participated in literature reviews and conceptualization. WC and S-IJ reviewed the manuscript and revised it. All authors reviewed the manuscript.
